# Evaluating efficacy and outcomes: comparison of laser treatment and crystallized phenol in pilonidal sinus disease

**DOI:** 10.3389/fsurg.2024.1494382

**Published:** 2025-01-06

**Authors:** Ahmet Cihangir Emral, Merter Gülen, Bahadır Ege

**Affiliations:** Department of General Surgery, Atılım University Faculty of Medicine, Ankara, Türkiye

**Keywords:** laser treatment, pilonidal sinus disease, phenol, minimally invasive surgery, postoperative outcome

## Abstract

**Objective:**

The aim of this study is to comprehensively evaluate the efficacy of laser ablation and crystallized phenol application in the treatment of pilonidal sinus disease, focusing on treatment success, recurrence rates, complications, and the patients' return to normal life.

**Material and method:**

Data from patients treated for pilonidal sinus disease with laser ablation and crystallized phenol application at our clinic between January 2020 and September 2023 were retrospectively reviewed. Preoperative data including pit counts, disease stage, preoperative pilonidal abscess history, disease duration (week), treatment success, recurrence/persistent disease, postoperative complications, healing time (days), and visual analogue scale (VAS) scores on postoperative days 1 and 7, as well as return to normal life (days), were analyzed.

**Results:**

A total of 121 patients were included in the study, with 51 receiving laser ablation and 70 receiving crystallized phenol application. The postoperative outcomes revealed that the wound healing period and postoperative VAS values were statistically significantly better in the laser ablation group.

**Conclusion:**

Wound healing was faster and postoperative pain was less in the laser group compared to the phenol group. According to this study, both methods can successfully treat the disease in selected cases.

## Introduction

Pilonidal sinus disease (PSD) is an inflammatory condition that typically occurs in the sacrococcygeal region (1). Although its etiology is not fully understood, it is generally considered an acquired condition (2). The widely accepted mechanism for the disease's etiology is the penetration of hairs shed from the head, neck, and back into the natal cleft, which initiates a foreign body reaction (3, 4).

PSD predominantly affects males aged 15–30 years, and risk factors include obesity, prolonged sitting, excessive body hair, a deep natal cleft, and genetic predisposition. Despite these risk factors, it appears that personal hygiene plays no significant role in the development of PSD. PSD can be asymptomatic or symptomatic. Symptoms may include discomfort or pain when sitting, a sensation of moisture or discharge in the natal cleft, and the formation of abscesses (5–7). Despite the identification of various treatment methods for PSD, including excision with primary closure, various flap techniques, endoscopic pilonidal sinus treatment (EPSIT), video-assisted ablation of the pilonidal sinus (VAAPS), crystallized phenol application, and laser ablation, no standard treatment has yet been established (8, 9).

The aim of this study is to comprehensively evaluate the efficacy of laser ablation and crystallized phenol application in the treatment of PSD, focusing on treatment success, recurrence rates, complications, and the patients' return to normal life.

## Materials and methods

Ethics committee approval number “47” dated 22.12.2023 was received from Atılım University Medicana Hospital for this study. Data from patients treated for PSD with laser ablation and crystallized phenol application at our clinic between January 2020 and September 2023 were retrospectively reviewed. Patients were followed up through regular clinical visits at our outpatient clinic. The first follow-up visit took place on postoperative day 1, and subsequent visits occurred at week 1, month 1, month 3, and month 12. During these visits, clinical examination was performed to assess wound healing and detect any complications. The data were obtained from prospectively standardized clinical notes. Informed consent was obtained from all individual participants included in the study.

Patients with recurrence, a history of inflammatory bowel disease, chronic steroid use, autoimmune disorders, chemotherapy/radiotherapy in the anorectal or sacrococcygeal region, concurrent malignancies, or diabetes were excluded from the study. This study included patients over the age of 18 who met the study criteria and were treated with laser ablation or crystallized phenol application for pilonidal sinus disease. Additionally, patients in stages I, IIa, and IIb of the Guner classification were included. The Guner clinical staging system for PSD (10) is presented in Table 1.

**Table 1:** The Guner clinical staging system for PSD (10).

Stage	Description
Stage I	Single pit in the midline, no lateral extension
Stage II	>1 pits in the midline, no lateral extension
Stage IIa	2–3 pits in the midline
Stage IIb	>3 pits in the midline
Stage III	Midline pit/pits plus lateral extension in one direction
Stage IV	Midline pit/pits plus lateral extension in both directions
Stage R	Recurrent PSD following any type of treatment

**Figure 1:**
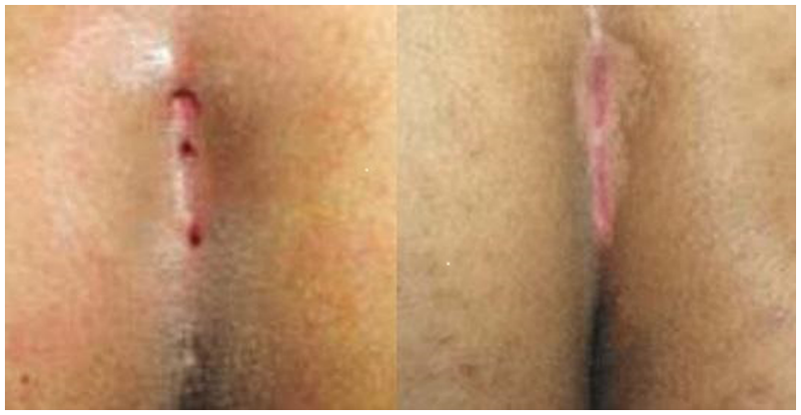
Complete healing after crystallized phenol application.

**Figure 2:**
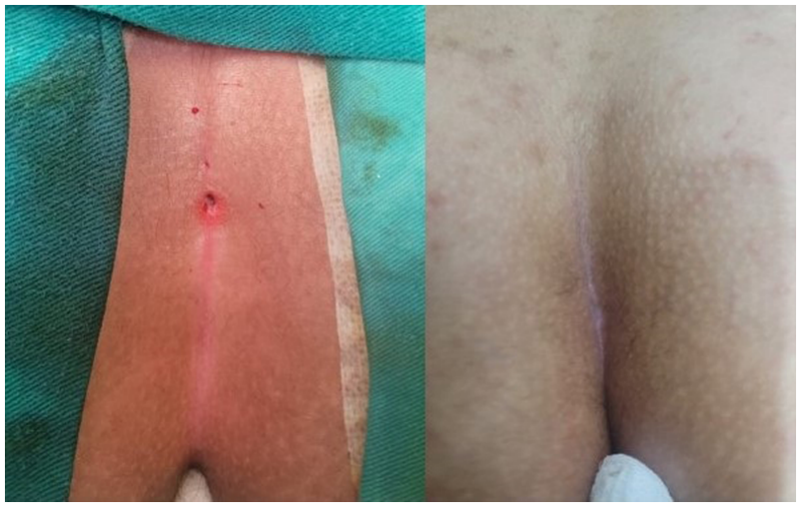
Complete healing after laser ablation.

Demographic information of the patients was recorded. All patients were followed up for a duration of one year. Data from preoperative and perioperative periods (operation day, postoperative day 1, week 1, month 1, month 3, and year 1) were obtained from examination notes. Complete healing was defined as the full epithelial closure of the sinus cavity as assessed by physical examination. Complete healing following the application of crystallized phenol is illustrated in Figure 1. Complete healing after laser ablation is shown in Figure 2. Patients who did not achieve epithelialization within 1 month were classified as having persistent cases. Recurrence was defined as the reappearance of an asymptomatic pit in the natal cleft or the development of an abscess or infection during the 1-year postoperative follow-up period in patients who had initially achieved complete healing.

Preoperatively, laser hair removal was recommended for all patients to address hair in the natal cleft area and, if present, in the back and neck regions. All patients underwent surgery in the prone position under sedation combined with local anesthesia (bupivacaine, 0.5 mg/kg). Preoperative antibiotic prophylaxis was administered with a single intravenous dose of 1 g cefazolin. All patients were discharged 4 h postoperatively. During the postoperative period, all patients received a 5-day course of antibiotic treatment (amoxicillin-clavulanic acid 2 × 1,000 mg).

Preoperative data including pit counts, disease stage, preoperative pilonidal abscess history, disease duration (week), treatment success, recurrence/persistent disease within 1 year, postoperative complications, healing time (days), and visual analogue scale (VAS) scores on postoperative days 1 and 7, as well as return to normal life (days), were analyzed. Patients treated with laser ablation were classified as Group L, and those treated with crystallized phenol were classified as Group P. Laser ablation and phenol application were administered to patients as a single treatment. Repetitive applications were not performed.

## Surgical technique

### Laser ablation

**Figure 3:**
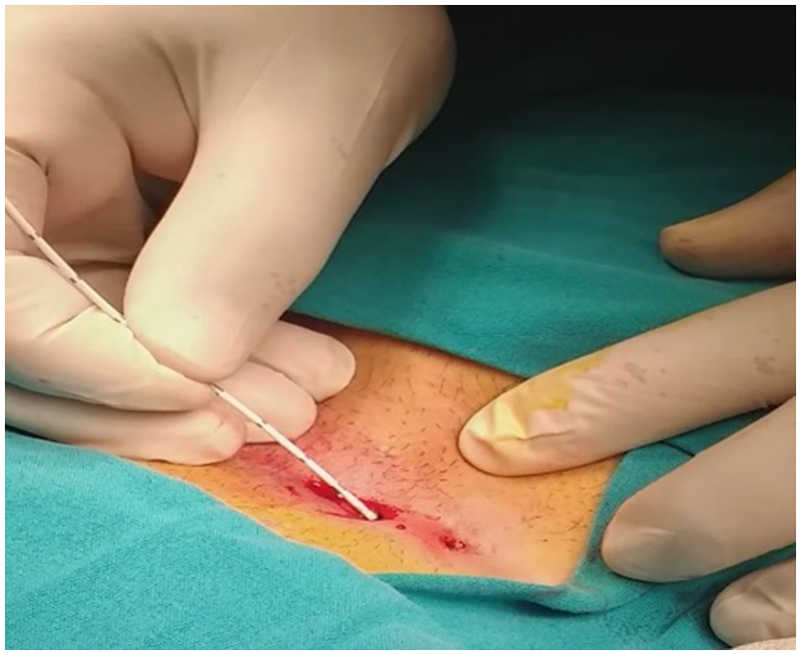
Application of laser ablation treatment in pilonidal sinus disease.

All pit orifices were dilated using a 2.8 mm punch biopsy needle. After cleaning the pit orifices and sinus cavity of hair and debris, curettage was performed. Then, using the NeoV V1470 Diode Laser (neoLaser Ltd, Caesarea, Israel) with a 2 mm probe, ablation was performed along each sinus tract at 10 W power, with a 5-s pulse duration, every 5 mm. Following ablation, sterile ice was applied to the pit orifice for 1 min, and then dressing was applied to complete the procedure. No sutures were used. Figure 3 shows the application of laser ablation treatment in pilonidal sinus disease.

### Crystallized phenol application

**Figure 4:**
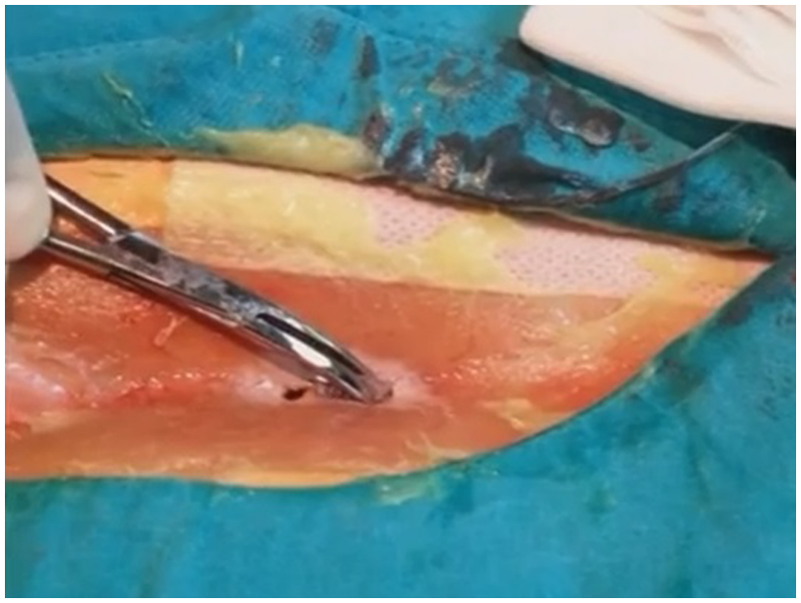
Application of crystallized phenol in pilonidal sinus disease.

All pit orifices were dilated using a 2.8 mm punch biopsy needle. After cleaning the pit orifices and sinus cavity of hair and debris, curettage was performed. Nitrofurazone 0.2% was applied around the pit to protect healthy skin. Subsequently, all pilonidal sinus cavities were filled with crystallized phenol (Phenol Crystalline; Botafarma, Ankara, Turkey) for 5 min. No sutures were used. Figure 4 shows the application of crystallized phenol in pilonidal sinus disease.

### Statistical analysis

Analyses were conducted using SPSS v27 (IBM-SPSS, Chicago, IL, USA). Distribution was assessed using skewness and kurtosis. Normally distributed data are presented as mean ± standard deviation (SD), and comparisons between two groups were performed using the independent-samples *t*-test. Non-normally distributed data are presented as median (range) and compared between two groups using the Mann–Whitney *U* test. Categorical variables were expressed as the number and percentage of patients and compared between groups using the chi-square test. A *p*-value of less than 0.05 was considered statistically significant.

## Results

A total of 121 patients were included in the study, with 51 receiving laser ablation and 70 receiving crystallized phenol application. The demographic and preoperative characteristics of the patients are shown in Table 2. No statistically significant differences were observed between the two groups in terms of age, gender, body mass index (BMI), history of previous abscesses, disease duration, number of pits, and disease stage.

**Table 2:** Demographic and preoperative characteristics of the patients.

Characteristics	Group L	Group P	*p*
Age, mean ± SD	25.9 ± 3.8	26.2 ± 4.3	0.62
Gender (%)			0.31
Female	6 (11.8)	13 (18.6)	
Male	45 (88.2)	57 (81.4)	
BMI, kg/m^2,^ mean ± SD	27.5 ± 1.9	28.3 ± 2.9	0.08
Previously Abscess History (%)	17 (33.3)	23 (32.9)	0.96
Disease duration, mean ± SD (week)	15.8 ± 6.5	14.3 ± 6.6	0.22
Number of pits, mean ± SD	1.9 ± 1.2	1.9 ± 1.1	0.97
Stage of disease (%)			0.27
Stage I	30 (58.8)	34 (48.6)	
Stage IIa	14 (27.5)	29 (41.4)	
Stage IIIb	7 (13.7)	7 (10)	
Stage III	0 (0)	0 (0)	
Stage IV	0 (0)	0 (0)	

The postoperative outcomes revealed that the wound healing period and postoperative VAS values were statistically significantly better in the laser ablation group. In the laser ablation group (Group L), 2 patients developed postoperative wound infections, while in the crystallized phenol group (Group P), 6 patients experienced similar infections. In these cases, infections were managed with daily local wound care using rifampicin 125 mg/2 ml for one week, and no abscesses developed. In Group L, recurrence of the disease was observed in 4 patients (7.8%), while in Group P, 7 patients (10%) experienced recurrence during the one-year follow-up. When comparing recurrence rates, no statistically significant difference was found between the groups (*p* = 0.76). In patients who experienced recurrence, flap techniques were performed. The postoperative outcomes of the patients are summarized in Table 3.

**Table 3:** Postoperative outcomes of the patients.

Postoperative outcomes	Group L	Group P	*p*
Recurrence (%)	4 (7.8)	7 (10)	0.76
Persistent Disease (%)	2 (3.9)	4 (5.7)	1
Postoperative Complication (%)	2 (3.9)	6 (8.6)	0.46
Wound Healing, days, median (min-max)	28 (20–54)	28 (20–56)	**0.01**
Postoperative Day 1 VAS value	1.4 ± 1.3	2.3 ± 1.1	**<0.01**
Postoperative Day 7 VAS value, median (min-max)	0 (0–4)	0 (0–3)	0.85
Return to normal activities, median (min-max)	2 (1–8)	2 (1–10)	0.89

The bold values indicate statistically significant results (*p* < 0.05).

## Discussion

Laser treatment eradicates the pilonidal sinus epithelium and induces contraction of the sinus cavity (7). In contrast, crystallized phenol destroys the epithelium through chemical irritation, leading to secondary healing of the sinus (11). In this study observed faster wound healing with laser ablation compared to crystallized phenol, which we attribute to the contraction of the sinus cavity induced by the laser, leading to a reduction in the cavity size.

Consistent with the literature, in this study found no significant superiority of either treatment option concerning postoperative complications and recurrence (9, 12, 13). Taşkın et al. (12) reported no significant difference in recurrence rates between laser and crystallized phenol treatments for pilonidal disease. It has been noted that patients with a higher number of pits or advanced stages of disease might experience higher recurrence rates with minimal invasive procedures, suggesting that more extensive surgical procedures might be recommended for these cases (10, 14). Additionally, patients who underwent drainage for pilonidal abscesses exhibited a higher risk of recurrence (15). Recurrences are typically observed within the first 9 months of treatment (14, 16). Considering the benefits of early return to daily life, low complications, and good cosmetic results, recurrence rates for both treatments seem acceptable. Furthermore, these methods can be reapplied in cases of recurrence. Pappas et al. found a 78.3% success rate when reapplying laser ablation to patients with recurrence (17). Similarly, Aygen et al. successfully treated 2 out of 5 patients with recurrence after phenol treatment by reapplying phenol (18). Stauffer et al. demonstrated in their meta-analyses that the application of laser and phenol increases the likelihood of recurrence in the long term. The fact that the follow-up period for patients in our study is only one year represents one of the most significant limiting factors of the study (19). While flap techniques are designed to reposition the natal cleft, we contend that addressing risk factors such as obesity, excessive hair growth, and prolonged sitting could further decrease recurrence rates. Johnson et al. (20) have suggested in their study that laser hair removal may be beneficial in reducing recurrence.

Regarding postoperative pain, in this study laser treatment resulted in less pain on postoperative day 1 compared to crystallized phenol. Despite the use of nitrofurazone 0.2% to protect the healthy skin during phenol treatment, chemical irritation caused by phenol at the sinus opening may occur. If this irritation occurs, it typically resolves within a few days with the use of topical treatments.

Conventional surgical methods involve the extensive removal of diseased skin and subcutaneous tissue, which can lead to large wounds and prolonged healing times (20). In contrast, minimally invasive techniques facilitate an earlier return to normal life due to the absence of subcutaneous drainage, reduced tissue excision, and lower rates of wound infections and complications. In both groups, the wound healing period did not adversely affect the return to normal activities, as patients were able to resume their usual activities relatively early in this study. Both laser ablation and phenol application have been shown to provide short hospital stays, early return to normal life, minimal postoperative pain, low complication rates, and good cosmetic results with acceptable recurrence rates in the literature (9, 19). In this study, no statistical difference was observed in postoperative complications, and wound infections were managed with daily wound care without abscess formation or the need for hospitalization.

Both methods provide better cosmetic results compared to conventional surgeries, which is particularly important for those who prioritize aesthetics. This factor likely plays a significant role in the preference for minimal invasive treatments. However, the potential health risks posed by phenol to both patients and healthcare providers should not be overlooked (21).

## Conclusion

Although wound healing was faster and postoperative pain was less in the laser group, both methods can successfully treat PSD in selected patient groups with good cosmetic outcomes, early return to normal life, acceptable recurrence rates, and low complication rates.

## Data Availability

The original contributions presented in the study are included in the article/Supplementary Material, further inquiries can be directed to the corresponding author.
